# Similarity-based methods for potential human microRNA-disease association prediction

**DOI:** 10.1186/1755-8794-6-12

**Published:** 2013-04-09

**Authors:** Hailin Chen, Zuping Zhang

**Affiliations:** 1 School of Information Science and Engineering, Central South University, Changsha, 410083, China; 2 Department of Computer Science and Technology, Hunan University of Humanities, Science and Technology, Loudi, 417000, China

**Keywords:** MicroRNA-disease association prediction, Network similarity, Network consistency

## Abstract

**Background:**

The identification of microRNA-disease associations is critical for understanding the molecular mechanisms of diseases. However, experimental determination of associations between microRNAs and diseases remains challenging. Meanwhile, target diseases need to be revealed for some new microRNAs without any known target disease association information as new microRNAs are discovered each year. Therefore, computational methods for microRNA-disease association prediction have gained a lot of research interest.

****Methods**:**

Herein, based on the assumption that functionally related microRNAs tend to be associated with phenotypically similar diseases, three inference methods were presented for microRNA-disease association prediction, namely MBSI (microRNA-based similarity inference), PBSI (phenotype-based similarity inference) and NetCBI (network-consistency-based inference). Global network similarity measure was used in the three methods to predict new microRNA-disease associations.

****Results**:**

We tested the three methods on 242 known microRNA-disease associations by leave-one-out cross-validation for prediction evaluation, and achieved AUC values of 74.83%, 54.02% and 80.66%, respectively. The best-performed method NetCBI was then chosen for novel microRNA-disease association prediction. Some associations strongly predicted by NetCBI were confirmed by the publicly accessible databases, which indicated the usefulness of this method. The newly predicted associations were publicly released to facilitate future studies. Moreover, NetCBI was especially applicable to predicting target diseases for microRNAs whose target association information was not available.

**Conclusions:**

The encouraging results suggest that our method NetCBI can not only provide help in identifying novel microRNA-disease associations but also guide biological experiments for scientific research.

## Background

Understanding the molecular mechanisms of diseases is an important goal in biomedical research. In this post-genomic era, numerous contributions [1-4], powered by advanced high-throughput genomic technologies, have been made towards this aim. Increasing evidence has revealed that microRNAs (miRNAs) play important roles in the development and progression of human diseases. An example reported recently is miR-518a. This dysregulated miRNA with some other miRNAs was discovered to be involved in the development of cervical carcinoma through controlling apoptosis signalling pathways and cell cycle regulation [5].

MiRNAs are a class of small non-coding RNAs typically between 19 and 22 nucleotides in length, which mainly repress the expression of target mRNAs at the post-transcriptional level by binding to the 3’-UTR of target mRNAs through sequence-specific base pairing, resulting in target mRNAs cleavage or translation inhibition [6-8]. In some cases miRNAs were also discovered to function as positive regulators [9,10]. Many investigators have reported that miRNAs are critical in tissue development [11], cell growth [12], cellular signalling [13], and so on. As such, the mutation of miRNAs, the dysfunction of miRNA biogenesis and the dysregulation of miRNAs and their targets may result to various diseases, such as lung cancer [14], lymphoma [15], breast cancer [16], and so on. These studies have produced a large number of miRNA-disease associations. Lu et al. [17] and Jiang et al. [18] manually retrieved the associations between miRNAs and diseases from literatures and constructed two curated databases, human miRNA-associated disease database (HMDD) and miR2Disease, respectively. They aim to offer comprehensive resources of experimentally confirmed miRNA-disease associations. Yang et al. [19] also created a publicly available database of Differentially Expressed MiRNAs in human Cancers (dbDEMC) with the goal to provide potential cancer-related miRNAs by *in silico* computing. However, the current knowledge about miRNA-disease associations is far from complete and experimental identification of miRNA-disease associations by genomic techniques is costly and time-consuming. Therefore there is a strong incentive to develop computational methods capable of detecting potential miRNA-disease associations effectively, through which further biological experiments can be guided.

Several computational approaches for miRNA-disease association prediction have been proposed based on the conclusions drawn by Lu et al. [17], who performed a comprehensive analysis to the human miRNA-disease association data and disclosed that miRNAs tend to show similar or different dysfunctional evidences for the similar or different disease clusters, respectively. Under the assumption that phenotypically similar diseases tend to be associated with functionally related miRNAs, Zhang et al. [20] used cardiovascular disease associated genes, miRNAs clusters, family analysis and Gene Ontology to develop a computational method to identify potential cardiovascular disease related miRNAs. A limitation of this method is that it has restricted application as the method ties to miRNAs sets. Jiang et al. [21] proposed a computational model based on the hypergeometric distribution to infer potential miRNA-disease associations by prioritizing the entire human microRNAome for diseases of interest. The notion that functionally related miRNAs tend to be associated with phenotypically similar diseases was reconfirmed in their manuscript. Although miRNA functional network, disease similarity network and known miRNA-disease associations were integrated in their work, only the neighbour information of each miRNA was used in their scoring system. Prediction accuracy would be increased by taking advantage of the global network similarity information. Another limitation is that *in silico* predicted associations were used as data sources in this method. It is known that these predicted associations used as data sources have some false-positive and false-negative results, thus influencing the final prediction accuracy. Chen et al. [22] adopted global network similarity measures and developed Random Walk with Restart for MiRNA-Disease Association (RWRMDA) to infer potential miRNA-disease associations by implementing random walk on the miRNA-miRNA functional similarity network. It was indicated in their work that global network similarity measures are better suited to capture the associations between diseases and miRNAs than traditional local network similarity measures. Good prediction performance was demonstrated in their experimental results. However, phenotype similarity information is not used in this method and RWRMDA does not work for diseases which do not have any known associated miRNAs. According to the assumption that miRNAs implicated in a specific tumor phenotype will show aberrant regulation of their target genes, Xu et al. [23] introduced an approach based on the miRNA target-dysregulated network (MTDN), constructed by combining computational target prediction with miRNA and mRNA expression profiles in tumor and nontumor tissues, to prioritize novel disease miRNAs. The drawback of this method is that negative samples are used, while there are no verified negative miRNA-disease associations in reality.

Computational prediction methods are important ways to choose the most promising miRNA-disease associations for further experimental examinations. The main difficulty of this task lies in the rarity of known miRNA-disease associations. In this paper, three inference methods, MBSI (microRNA-based similarity inference), PBSI (phenotype-based similarity inference) and NetCBI (network-consistency-based inference), were introduced to predict potential miRNA-disease associations based on the global network similarity measure and the assumption that functionally related miRNAs tend to be associated with phenotypically similar diseases. MiRNA functional similarity network, disease similarity network and known miRNA-disease associations were integrated in our work. For the three methods, each miRNA-disease association was scored and high prediction scores could be expected to have high probabilities of miRNA-disease associations. Benchmark dataset with known miRNA-disease associations was used to assess the performance of our proposed methods. The best-performed method NetCBI was then selected for potential miRNA-disease association prediction. Some predicted associations with high-ranks were manually checked and were confirmed from the publicly available databases. We take these as strong evidence to support the practical application of our approach. Our comprehensively predicted miRNA-disease associations also enable us to suggest many potential miRNA-disease associations, which can offer help in further experiments and hence increase research productivity.

## Results

### MiRNA-disease association network construction and analysis

**Figure 1:**
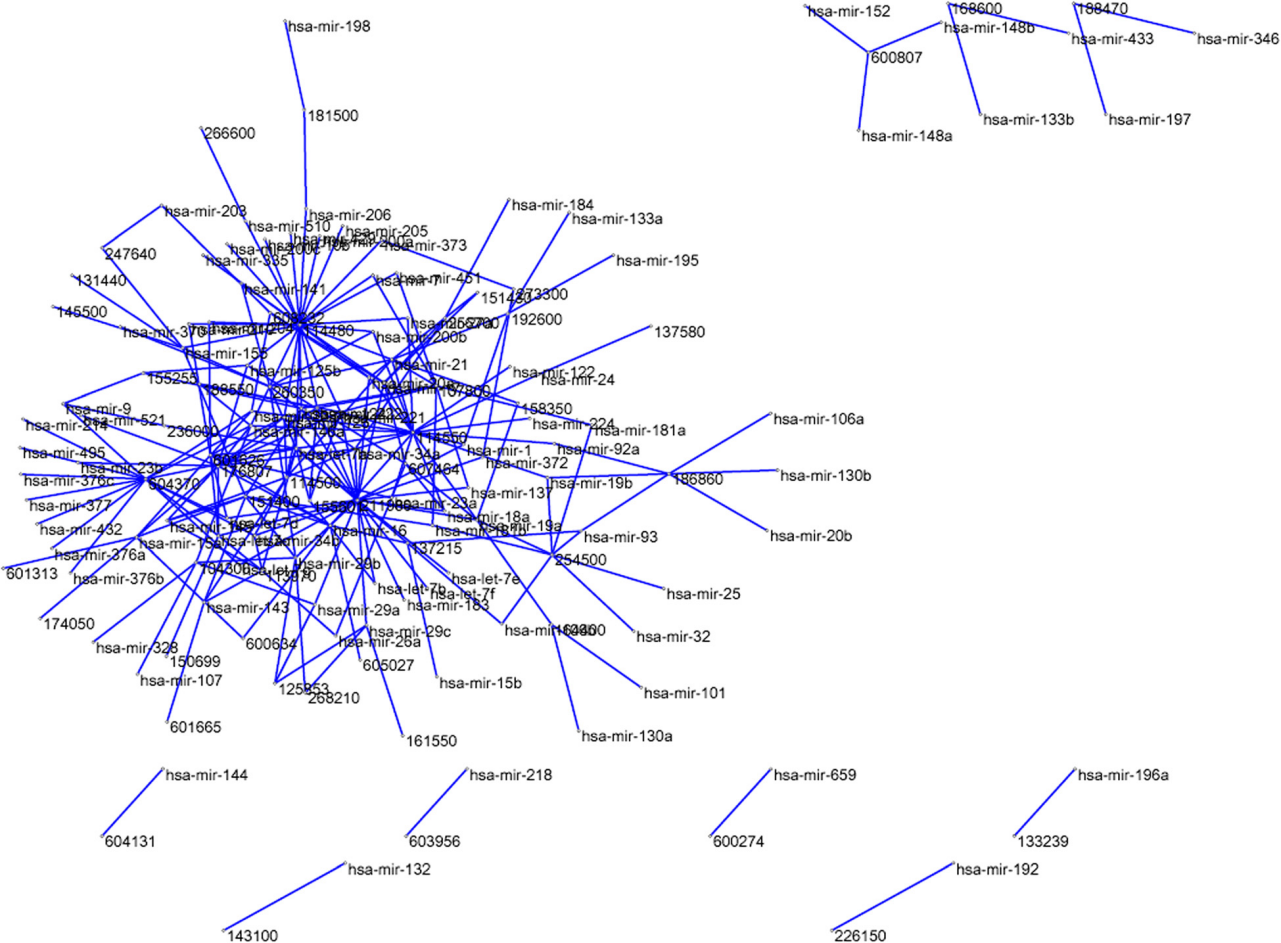
**MiRNA-disease phenotype network (MP network).** The MP network is generated by using 242 experimentally verified associations between miRNAs and diseases. The network is prepared by Pajek (http://vlado.fmf.uni-lj.si/pub/networks/pajek/).

In this study, we first focus on the verified miRNA–disease associations. The set of 242 known miRNA–disease associations (see Methods) is regarded as the ‘gold standard’ data, and is used for evaluating the performance of our proposed methods in the cross-validation experiments as well as training data in the comprehensive prediction. We constructed the miRNA–disease association network using a bipartite graph representation (see Figure 1) and analysed some statistics for the miRNA-disease association network. In the bipartite graph, the heterogeneous nodes correspond to either miRNAs or diseases, and edges correspond to associations between them. An edge is placed between a miRNA node and a disease node if the disease is known to associate with the miRNA.

**Figure 2:**
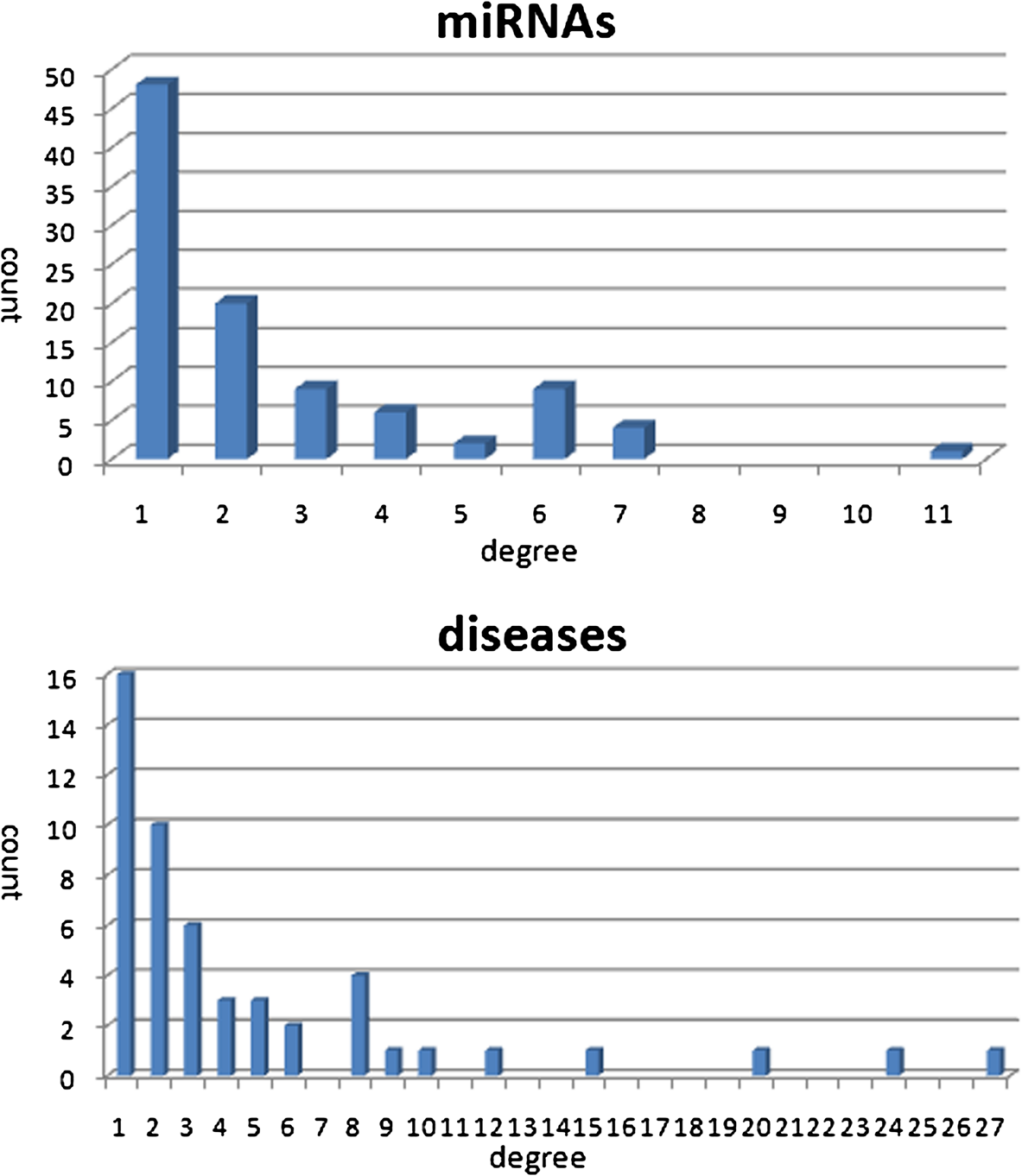
**Degree distributions for miRNAs and diseases in the miRNA-disease phenotype network.** The top panel shows the histograms of the degree distributions of miRNAs. The bottom panel shows the histograms of the degree distributions of diseases.

Figure 2 shows the degree distributions for miRNAs and diseases in the miRNA–disease association network. The degree of the miRNA (respective disease) node is the number of diseases that the miRNA has associations with (respectively the number of miRNAs targeting the disease).

Table 1 details some statistics for the miRNA-disease association network, such as average degree of miRNAs and average degree of diseases. Inspection of the miRNA–disease association network shows that the miRNAs and their target diseases tend to be densely clustered, while it also comprises a few small unconnected components.

**Table 1:** Statistics for the miRNA-disease association network

**No. of miRNAs**	**No. of diseases**	**No. of miRNA-disease associations**	**Average degree of miRNAs**	**Average degree of diseases**
99	51	242	2.44	4.75

### Performance evaluation of the proposed methods

The three methods, MBSI, PBSI and NetCBI, were tested on the 242 known miRNA-disease associations to assess their power to infer potential miRNA-disease associations. We performed a leave-one-out cross-validation on each method. For PBSI, the miRNA associations of each query disease were left out once as the testing case. For MBSI and NetCBI, the associations between a query miRNA and all its disease phenotypes including the target disease phenotype(s) were removed in the leave-one-out cross-validation. We prioritized the entire associations according to the scores derived from the three scoring systems.

**Figure 3:**
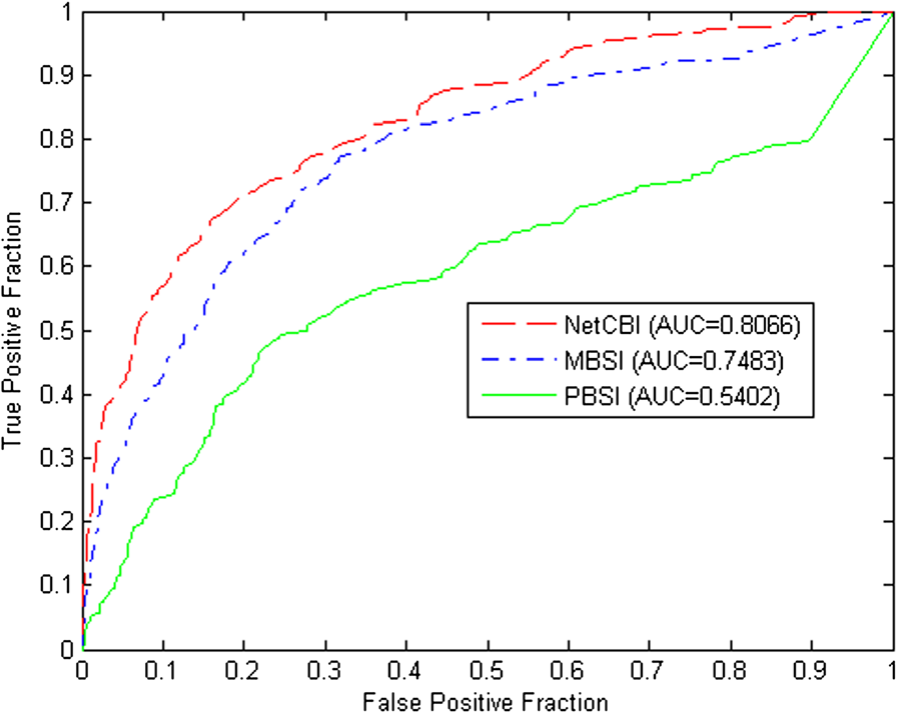
ROC curves and AUC values of the three proposed methods to predict miRNA-disease associations in the benchmark dataset by leave-one-out cross-validation tests.

We calculated the sensitivity and specificity for each threshold. Sensitivity refers to the percentage of the associations whose ranking is higher than a given threshold, namely the ratio of the successfully predicted experimentally verified miRNA-disease associations to the total experimentally verified miRNA-disease associations. Specificity refers to the percentage of associations that are below the threshold. A receiver-operating characteristics (ROC) curve was plotted by varying the threshold, and the value of area under curve (AUC) was calculated. Take NetCBI as an example. The values of all disease associations of one miRNA are available after one round of leave-one-out cross validation. Each value is taken as a threshold for calculating true positive fraction (TPF) and false positive fraction (FPF).Then ROC curve is plotted and AUC value is calculated according to these TPFs and FPFs. We finally report the average AUC values of the three methods. Figure 3 shows the ROC curves and average AUC values of our three inference methods for miRNA-disease association prediction. For NetCBI, the result produced by the best parameters in the leave-one-out cross-validation was reported. When our methods were tested on the 242 experimentally verified miRNA-disease associations, three AUC values of 74.83%, 54.02% and 80.66% were achieved, suggesting that the two methods, MBSI and NetCBI, can recover the known experimentally-verified miRNA-disease associations, and therefore have the potential to infer new miRNA-disease associations.

### Effects of parameters in NetCBI

There are two parameters in our method NetCBI. To investigate the selection of the two parameters for the performance of NetCBI, we set various values for them and calculated the AUC values in the framework of leave-one-out cross-validation. Additional file 1 details the effects of the two parameters on the cross validation results in the benchmark dataset. After a comprehensive searching, the parameters (*α=*0.1*, β=*0.1) led to best AUC result are selected for performance comparison and further association prediction.

To get an unbiased estimate, we conducted a nested leave-one-out cross validation in NetCBI. We split the 99 miRNA samples into three parts-97 miRNAs for training, 1 miRNA for test and 1 miRNA for validation. Parameter optimization is conducted within the 98 miRNAs (97 miRNAs for training and 1 miRNA for test) and performance evaluation is based on the validation part. Leave-one-out cross validation was conducted in each inner loop for parameter optimization, which included 98 iterations. The outer loop of performance evaluation was also based on leave-one-out cross validation, which included 99 iterations. We finally received a slightly reduced AUC value of 79.77%, with parameters *α=*0.2618 ± 0.01*, β=*0.2618 ± 0.01. It can be observed that the results received in NetCBI are quite robust to parameter changes.

### Comparison with other methods

Until recently, several computational methods have been proposed for miRNA-disease association prediction. Different models have been constructed based on different data features, such as Gene Ontology, miRNA function similarity value, miRNAs clusters, and so on, which makes performance comparison difficult. An AUC value of 75.80% was achieved under the assumption of the hypergeometric distribution for prioritizing miRNAs in [21]. When this model was applied to diseases without any known related miRNAs, a reduced AUC value of 69.51% was obtained. Unlike the method presented in [21], our proposed methods make full use of global network similarity measures, including miRNA-miRNA functional similarity and disease phenotype similarity, and the best-performed approach NetCBI received a higher AUC value of 80.66%. Although another higher AUC value of 86.17% was achieved in [22], phenotype similarity information was not used in this method and it was not applicable to diseases without any known related miRNAs.

### Comprehensive prediction for unknown miRNA-disease associations

**Figure 4:**
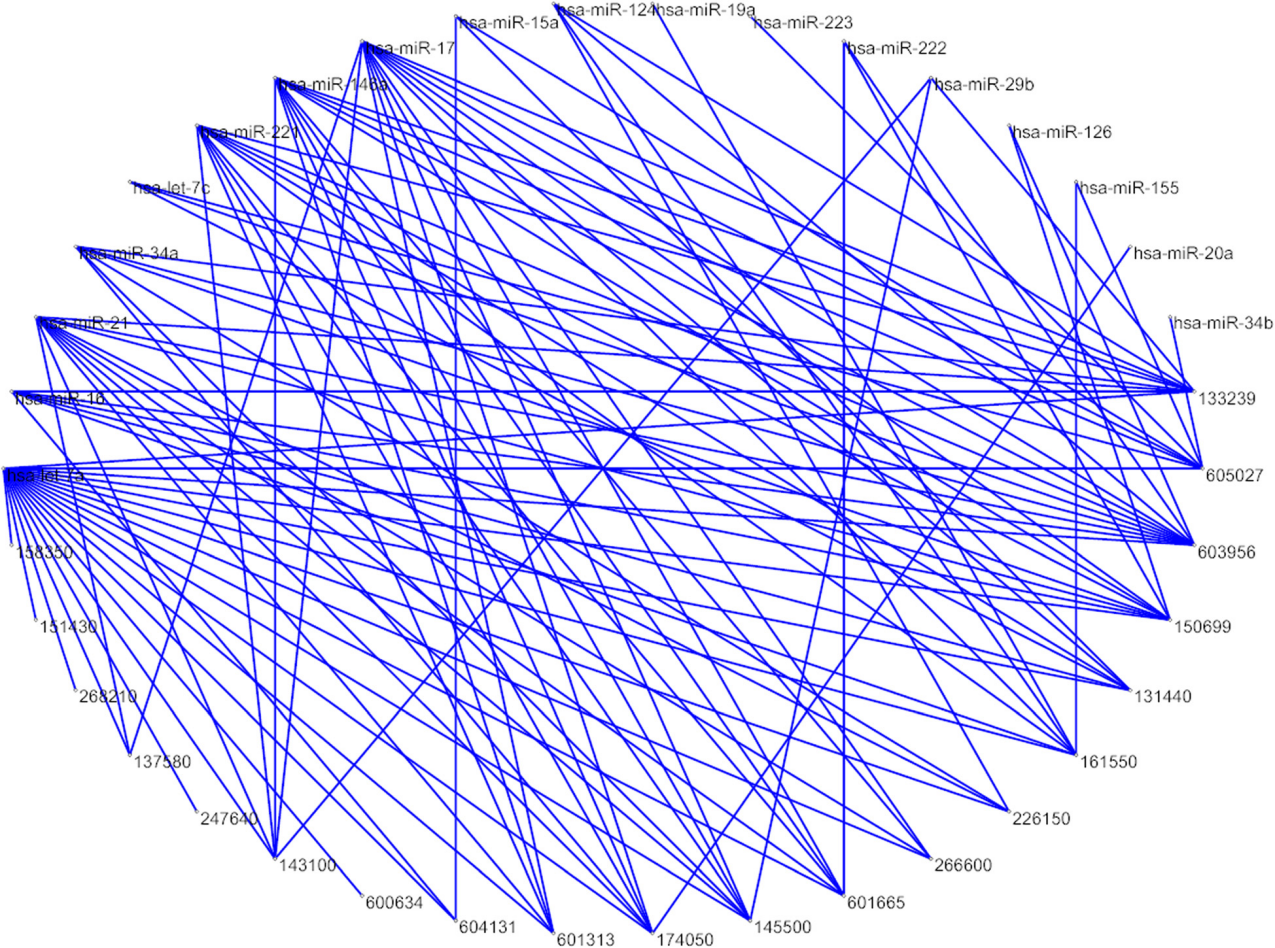
**Newly predicted miRNA-disease association network with the top 100 highest scores.** The network is prepared by Pajek (http://vlado.fmf.uni-lj.si/pub/networks/pajek/).

After confirming the usefulness of our methods, we chose the best-performed method NetCBI to conduct a comprehensive prediction of unknown associations between all possible miRNAs and diseases. In the inference process for these predictions, we trained NetCBI with all the known associations. Parameters *α* and *β* are set to be 0.1. We ranked the non-associating pairs with respect to their association scores. The prediction results for unknown miRNA-disease associations with the top 100 highest scores are shown in Figure 4. The full list of the top 100 prediction results can be obtained from the Additional file 2. Furthermore, we manually checked the top 10 predicted associations from the latest online versions of HMDD [17], miR2Disease [18] and dbDEMC [19] databases. We confirmed that 6 associations (Table 2) are now annotated in at least one of the three databases. Meanwhile a *p*-value of 0.006 is received using Fisher's exact test. We take these as strong evidence to support the practical application of our approach. Note that the predicted associations that are not reported yet may also exist in reality.

**Table 2:** The newly confirmed miRNA-disease associations in the top 10 predicted results by NetCBI

**miRNA**	**OMIM Disease ID**	**Rank**	**Source**
hsa-let-7a	133239	1	HMDD
hsa-let-7a	605027	2	HMDD
hsa-let-7a	603956	3	dbDEMC
hsa-let-7a	150699	4	
hsa-let-7a	131440	5	HMDD
hsa-let-7a	161550	6	HMDD
hsa-let-7a	226150	7	
hsa-let-7a	266600	8	HMDD
hsa-miR-16	161550	9	
hsa-miR-21	150699	10	

## Discussion

The current difficulties of developing computational methods for the prediction of miRNA-disease associations lie in three aspects. Firstly, the known miRNA-disease associations are rare. Secondly, negative samples are hard or even impossible to select as there are no verified negative miRNA-disease associations. Thirdly, association prediction should also be made to miRNAs without any known target disease association information as new miRNAs are discovered each year.

Here, we presented three computational methods for the prediction of miRNA-disease associations. All the three methods do not use negative samples. The essential difference of the three methods is the definition of similarity. MBSI is based on miRNA functional similarity, and PBSI is based on phenotype similarity, whereas NetCBI is based on both of the two similarity values. Based on the foundations of previous research [24,25], the best-performed method NetCBI focuses on improving detection of miRNA-disease associations by integrating the miRNA functional similarity information and the human disease similarity information to better summarize sparse associations for a global comparison of all possible miRNA-disease associations. The global relevance between a query miRNA and all the miRNAs is measured with graph Laplacian scores in NetCBI. The global relevance between a target disease and all disease phenotypes is similarly calculated. NetCBI uses information in the miRNA network and the disease network simultaneously to analyze associations between miRNAs and diseases.

Comparison among the three proposed methods indicated that integration of miRNA function similarity value and disease phenotype similarity value can improve prediction performance. The worst AUC value of PBSI on the benchmark dataset indicated that prediction based on phenotype similarity alone was poor. Compared with some existing methods that also utilized the miRNA functional similarity information and the human disease similarity information, NetCBI is more flexible in handling the association prediction because NetCBI is able to predict disease phenotypes for new miRNAs whose target disease association information is not available. This feature is very useful because new miRNAs are discovered each year and their target diseases need to be revealed.

One previous research related with this study is the prioritization of disease miRNAs based on the hypergeometric distribution [21], but only the neighbour information of each miRNA was used, which limited its prediction accuracy. The most recent study related with our work is miRNA-disease association inference based on random walk on a miRNA-miRNA functional similarity network [22]. However, phenotype similarity information was not taken into consideration, and newly detectable associations were limited to diseases with known associated miRNAs.

Despite the encouraging results of NetCBI, there are also limitations. NetCBI depends heavily on network similarity measure, and the known experimentally verified miRNA-disease associations were rare. Therefore, integrating other bioinformatics sources, such as Gene Ontology, might improve model performance. From a technical viewpoint, the performance of our method could be improved by using more accurate similarity information designed for miRNAs and diseases.

## Conclusions

We presented three similarity-based methods to predict associations between miRNAs and human diseases. We took advantage of both OMIM phenotype similarity information and miRNA functional similarity information in the best-performed method NetCBI. Best performance among the three methods and further confirmation of some strongly-predicted miRNA-disease associations in publicly accessible databases indicate the realistic application of NetCBI. The top 100 potential miRNA-disease associations predicted by NetCBI are released publicly to facilitate biological experiments for the contribution to the identification of true miRNA-disease associations. The methods we proposed will be an important bioinformatics resource in biomedical research to identify the roles of miRNAs in human diseases.

## Methods

### Data sources

The benchmark dataset (see Additional file 3) used in this manuscript is downloaded from [21,26,27]. Here below we provide a brief description.

### The miRNA-miRNA functional similarity data

The miRNA-miRNA functional similarity scores were downloaded from http://cmbi.bjmu.edu.cn/misim/[26]. In this dataset, a functional similarity score for each miRNA pair is calculated based on the observation that genes with similar functions are often associated with similar diseases. The miRNA functional similarity scores have been successfully used to infer novel human miRNA-disease associations in [22].

### The disease phenotype similarity data

We downloaded the disease phenotype similarity scores from the MimMiner [27], developed by van Driel et al. who computed a phenotype similarity score for each phenotype pair by the text mining analysis of their phenotype descriptions in the Online Mendelian Inheritance in Man (OMIM) database [28]. The phenotypic similarity scores have been successfully used to predict or prioritize disease related protein-coding genes [29,30].

### The human miRNA-disease association data

We downloaded the 270 known experimentally verified miRNA-disease associations provided in [21]. We discovered that 19 miRNAs could not be searched in [26]. After removing the 19 miRNAs from the 270 known associations, we finally received 242 verified miRNA-disease associations consisting of 99 miRNAs and 51 disease phenotypes.

### Method description

We denote the miRNA set as *M* = {*m*_1_, *m*_2_, …, *m*_*n*_} and the phenotype set as *P* = {*p*_1_, *p*_2_, …, *p*_*m*_}, the miRNA-disease associations can be described as a bipartite MP graph *G*(*M*, *P*, *E*), where *E* = {*e*_*ij*_ : *m*_*i*_ ∈ *M*, *p*_*j*_ ∈ *P*}. A link is drawn between *m*_*i*_ and *p*_*j*_ when the miRNA *m*_*i*_ is associated with the phenotype *p*_*j*_. The MP bipartite network can be presented by an *n×m* adjacent matrix {*a*_*ij*_}, where *a*_*ij*_*=*1 if *m*_*i*_ and *p*_*j*_ is linked, while all other unknown miRNA-disease pairs are labeled as 0 to indicate they are going to be predicted. We define *M*(n*n), *P*(m*m), and *a*(n*m) as the adjacency matrix of the miRNA functional similarity network, the disease phenotype similarity network, and the miRNA-disease association network, respectively.

#### MicroRNA-based similarity inference (MBSI)

The basic idea of this method is: if a miRNA is associated with a disease, then other miRNAs similar to the miRNA will be recommended to be associated with the disease. For an MP pair *m*_*i*_*-p*_*j*_, a linkage between *m*_*i*_ and *p*_*j*_ is determined by the following predicted score:

(1) $$v_{\mathrm{ij}}^{P}=\frac{\sum_{l=1,l\neq i}^{n}S\left(m_{i},m_{l}\right)a_{\mathrm{lj}}}{\sum_{l=1,l\neq i}^{n}S\left(m_{i},m_{l}\right)}$$

where *S*(*m*_*i*_, *m*_*l*_) is miRNA functional similarity value between miRNAs *m*_*i*_ and *m*_*l*_.

#### Phenotype-based similarity inference (PBSI)

The basic idea of this method is: if a miRNA is associated with a disease, then the miRNA will be recommended to be associated with other similar diseases. For an MP pair *m*_*i*_*-p*_*j*_, a linkage between *m*_*i*_ and *p*_*j*_ is determined by the following predicted score:

(2) $$v_{\mathrm{ij}}^{M}=\frac{\sum_{l=1,l\neq j}^{m}S\left(p_{j},p_{l}\right)a_{\mathrm{il}}}{\sum_{l=1,l\neq j}^{m}S\left(p_{j},p_{l}\right)}$$

Where *S*(*p*_*j*_, *p*_*l*_) is disease phenotype similarity value between diseases *p*_*j*_ and *p*_*l*_.

#### Network-consistency-based inference (NetCBI)

The basic idea of network consistency is that, if miRNAs are ranked by their relevance to a query miRNA, and phenotypes are ranked by their relevance to the hidden target phenotype of the query miRNA, the top-ranked miRNAs and the top-ranked disease phenotypes should be highly connected by known associations. Unlike the above two inference methods, NetCBI integrates the miRNA-miRNA functional similarity network data and the disease phenotype similarity network data. The idea of network consistency has been successfully used to predict gene-phenotype associations in [24]. The solid foundation for the algorithm can be traced back to [25]. We formulate a graph query problem for miRNA and disease association discovery. The query miRNA is represented by a binary vector *m* = [*m*_1_, *m*_2_, …, *m*_*n*_]^*T*^ denoting the miRNA membership against the miRNA set, i.e. each *m*_*i*_*=*1 if miRNA *i* is the query miRNA, otherwise *m*_*i*_*=*0. Similarly, the list of target phenotypes is given by another binary vector *p* = [*p*_1_, *p*_2_, …, *p*_*m*_]^*T*^ and phenotype *j* is a target phenotype if *p*_*j*_*=*1.

To make full use of global network similarity information, we compute the global relevance score between the query miRNA *m* and all the miRNAs based on the graph Laplacian of the miRNA functional similarity network *M*(n*n). We first normalize *M* as $\bar{M}=M\left(:,i\right)/\mathrm{sum}\left(M\left(:,i\right)\right)$, where *i* is the column number of *M*. A vector $\tilde{m}$ of graph Laplacian scores is derived from:

(3) $$\min_{\tilde{m}}\sum_{i,j}\overset{―}{M_{i,j}}{\left(\tilde{m_{i}}-\tilde{m_{j}}\right)}^{2}+\frac{1-\alpha }{\alpha }\underset{i}{\sum }{\left(\tilde{m_{i}}-m_{i}\right)}^{2}$$

In Equation (3), the first term is a smoothness penalty, which forces connected miRNAs to receive similar scores, and the second term ensures the consistency with the query miRNA. Parameter *α* ∈ (0, 1) balances the contributions from the two penalties. The close solution to Equation (3) is

(4) $$\tilde{m}=\left(1-\alpha \right){\left(I-\alpha \overset{―}{M}\right)}^{-1}m$$

Similarly, graph Laplacian scores can be derived to measure the relevance between the phenotypes and the target phenotype *p* with the close solution

(5) $$\tilde{p}=\left(1-\beta \right){\left(I-\beta \overset{-}{P}\right)}^{-1}p$$

where $\bar{P}$ is the normalized *P* and parameter *β* ∈ (0, 1).

Our method uses consistency in networks to measure whether the query miRNA *m* and a target phenotype *p* show coherent association with the known miRNA-phenotype associations. Specifically, given the graph Laplacian scores *m*, which ranks the miRNAs by their relevance to the query miRNA $\tilde{m}$, and the graph Laplacian scores $\tilde{p}$, which ranks the phenotypes by their relevance to the hidden target phenotype *p*, NetCBI measures whether the associations given by *a* are connecting miRNAs and phenotypes with similar scores in $\tilde{m}$ and $\tilde{p}$. We simply go through each phenotype and compute a Pearson correlation coefficient score against the query miRNA *m* for each case.

(6) $$\mathrm{NetCB}I_{\mathrm{corr}}\left(\tilde{m},\tilde{p},a\right)=\mathrm{corr}\left(a\tilde{p},\tilde{m}\right)$$

Finally, the phenotype(s) with the highest score(s) is chosen as the target phenotype(s).

## Competing interests

The authors declare that they have no competing interests.

## Authors’ contributions

HC and ZZ conceived and designed the experiments. HC performed the experiments. HC and ZZ analyzed the data. HC and ZZ wrote the paper. Both authors read and approved the final manuscript.

## Acknowledgements

We are grateful to Dr. Yixiong Liang at Central South University for useful discussions. We thank Dr. Qinghua Cui from Peking University Health Science Center, Prof. Yi Pan of Georgia State University, Prof. Jianxin Wang at Central South University, Prof. Yadong Wang of Harbin Institute of Technology and Dr. Assaf Gottlieb at Stanford University for their help. This research was supported by the National Natural Science Foundation of China (grant 60970095, grant 61003124 and grant M1121008), Research Fund for the Doctoral Program of Higher Education of China (Grant No. 20120162110077), the National High Technology Research and Development Program of China (863 Program, No.2012AA011205) and the Program for New Century Excellent Talents in University(NCET-12-0547).

## Authors’ contributions

HC and ZZ conceived and designed the experiments. HC performed the experiments. HC and ZZ analyzed the data. HC and ZZ wrote the paper. Both authors read and approved the final manuscript.

## Pre-publication history

The pre-publication history for this paper can be accessed here:

http://www.biomedcentral.com/1755-8794/6/12/prepub

## Supplementary Material

Additional file 1 The effects of two parameters in NetCBI. Click here for file

Additional file 2 **100 top-ranked potential miRNA-disease associations.** Each line represents a potential association between miRNAs and human diseases, including miRNA ID, OMIM ID and OMIM name. Click here for file

Additional file 3 The benchmark dataset used in this manuscript. Click here for file
